# Monochromatic higher order aberrations in highly myopic eyes with Staphyloma

**DOI:** 10.1186/s12886-021-01965-9

**Published:** 2021-05-18

**Authors:** Santiago Delgado-Tirado, Alberto López-Miguel, Yazmin Báez-Peralta, Lucía González-Buendía, Itziar Fernández, Jorge L. Alió, Miguel J. Maldonado, Rosa M. Coco-Martín

**Affiliations:** 1 Instituto de Oftalmobiología Aplicada (IOBA), Universidad de Valladolid, Valladolid, Spain; 2 Networks for Cooperative Research in Health (Oftared), Instituto de Salud Carlos III, Madrid, Spain; 3 Division of Ophthalmology, School of Medicine, Universidad Miguel Hernández, Alicante, Spain

**Keywords:** Dome-shaped macula, High myopia, Higher order aberrations, Staphyloma

## Abstract

**Background:**

Prevalence of high myopia is continuously increasing, thus, patients affected with staphyloma are abundant worldwide. Assessment of the quality of vision in these patients is mandatory for a proper clinical counselling, specially when undergoing surgical procedures that require intraocular lenses implantation. Thus, the purpose of the study was to assess monochromatic higher order aberrations (HOAs) in highly myopic eyes with staphyloma with or without a dome-shaped macula.

**Methods:**

Participants underwent spectral-domain optical coherence tomography, ocular axial biometry, dual Scheimpflug photography and integrated Placido disk topography, and Hartmann-Shack wavefront analysis. Five groups were evaluated: a low-moderate myopia control group (< 6.00 diopters, *n* = 31) and four high myopia (≥6.00 diopters) groups: eyes without staphyloma (*n* = 18), eyes with inferior staphyloma (*n* = 14), eyes with posterior staphyloma without dome-shaped macula (*n* = 15) and eyes with posterior staphyloma with dome-shaped macula (*n* = 17). Subsequently, two new groups (including all participants) were created to assess differences between myopia with and without staphyloma. One-way analysis of covariance was performed using age and lens densitometry as covariates.

**Results:**

Statistically significant (*p* ≤ 0.05) differences in anterior corneal fourth-order HOAs were observed between the low-moderate myopia and no-dome-shaped macula (Mean: 0.16 μm) and dome-shaped macula posterior staphyloma groups (Mean: 0.12 μm) in younger patients (≤45 years old). The same groups also showed (*p* ≤ 0.05) significant differences for anterior corneal primary spherical aberration (Mean: 0.19 and 0.13 μm, respectively). In addition, anterior corneal tetrafoil was significantly higher (*p* = 0.04) in dome-shaped macula compared to no-dome-shaped macula (Mean: 0.18 vs 0.06 μm, respectively). When all participants were grouped together, significantly lower mean anterior corneal primary spherical aberration (0.15 μm vs. 0.27 μm, *p* = 0.004) and higher internal primary spherical aberration (0.04 μm vs. -0.06 μm, *p* = 0.04) was observed in staphyloma compared to no-staphyloma myopic patients.

**Conclusions:**

Eyes with high myopia and staphyloma have less positive anterior corneal primary spherical aberration and less negative internal primary spherical aberration, suggesting that the anterior corneal surface tends to mimic in a specular fashion the posterior pole profile. This corneal behaviour appears to change in patients older than 45 years.

**Supplementary Information:**

The online version contains supplementary material available at 10.1186/s12886-021-01965-9.

## Background

Uncorrected myopia is a major cause of visual impairment and the second cause of blindness globally [1]. Its prevalence varies depending on the geographic area, and more importantly, it is increasing worldwide [1]. It is estimated that in 2050, 50% of the world population will be myopic, and 10% will have high myopia (HM) [1], defined as myopia of − 6.00 diopters (D) or higher or an ocular axial length of 26 mm or more [2]. This trend indicates 2-fold and 5-fold increases, respectively, from the current myopia prevalence [1].

Wavefront aberrations are a significant cause of degradation of retinal image quality [3, 4]. It is well-known that ocular aberrations increase with age [3, 5, 6]. Several studies have reported the increase in ocular higher order aberrations (HOAs) with increasing axial length [7–10]. HM patients now not only require good visual acuity but also good quality of vision. These patients also are increasingly undergoing refractive surgery procedures including phakic and pseudophakic intraocular lens implantation and demanding optimal quality of vision [11].

Interestingly, most research addressing the relationship between HOAs and axial length is focused on the study of anterior corneal and ocular HOAs [9, 12], but the information about the internal HOAs in HM is limited. The lens in phakic eyes is the main source of internal HOAs [13], thus, it is critical to have reliable measurements in order to improve predictability and reliability of the results. However, staphyloma is one of the most frequent findings in HM that can be accompanied by macular bending due to both an inferior staphyloma whose border crosses the macular area or to a dome-shaped macula in an eye with posterior staphyloma. We hypothesize that this anatomic variation found in HM patients [14] could critically affect current refraction and HOAs measurements impacting the final quality of vision of patients undergoing refractive surgery procedures. Considering the growing number of population with HM, staphyloma is more likely to be found. However, few studies have been published on the effect of staphyloma on the HOAs measurements [10, 15], and no studies of cases with macular bending due to inferior staphyloma and/or dome-shaped macula. The goal of the current study was to comprehensively assess monochromatic anterior corneal, internal, and ocular HOAs in HM eyes with staphyloma.

## Methods

A prospective cross-sectional clinical study was performed to analyse myopic and highly myopic patients.

### Participants

The inclusion criteria for the case eyes (HM) was a myopic refractive error of 6.00 D or more, and less than 6.00 D for low-moderate myopia control eyes. The exclusion criteria were the presence of media opacities (i.e., corneal haze or cataract), refractive astigmatism over 1.00 D, corneal ectasia, active anterior (i.e., keratitis) and/or posterior segment anomalies except for staphyloma, and a history of previous ocular surgery or ocular trauma. All participants had to belong to the same ethnic group to avoid bias. The Caucasian type was chosen because in Spain the vast majority of the population is Caucasian. All participants had a comprehensive ocular examination including manifest refraction, ocular biometry, a detailed ophthalmoscopic evaluation and optical coherence tomography imaging. In the present study, posterior staphyloma was defined like Spaide did as “an outpouching of the wall of the eye that has a radius of curvature that is less than the surrounding curvature of the wall of the eye“ [16]. Dome-shaped macula was defined as unexpected inward bulge of the macular area that can be accurately identified in optical coherence tomography images [17]. If participants showed a bending involving the macular area (observed in optical coherence tomography images) due to the presence of an inferior staphyloma [14], they were classified into the inferior staphyloma group.

Participants were recruited from the IOBA-Eye Institute outpatient clinic. A total of five groups were created for this study: four HM case groups and one low-moderate myopia control group were created initially based on the refractive error and ocular characteristics evaluated by indirect ophthalmoscopy and spectral-domain optical coherence tomography (Topcon Corp., Tokyo, Japan) examinations. The low-moderate myopia control group was comprised of healthy eyes with a refractive error below 6.00 D of myopia, HM group 1 included HM eyes (myopia ≥6.00 D) without staphyloma, HM group 2 included HM eyes (myopia ≥6.00 D) with inferior staphyloma, HM group 3 included HM eyes (myopia ≥6.00 D) with posterior staphyloma without a dome-shaped macula, and HM group 4 included HM eyes with posterior staphyloma and a dome-shaped macula.

The total sample size calculated was 64, at least 13 per group, based on 80% power with a 5% significance level and a large effect size for an analysis of covariance study (1 covariable) that included five groups. The participants were recruited consecutively for all groups and it was completed only when a sample size of 14 was reached in all groups.

In order to assess if there were differences in HOAs between myopic eyes with or without staphyloma, all participants were also classified into two new groups: eyes with and without staphyloma. The no-staphyloma group was comprised of low-moderate myopia and HM eyes without staphyloma; and the staphyloma group was comprised of HM eyes with inferior or posterior staphyloma.

### Procedures

Participants were evaluated using an integrated Placido disk topography and Hartmann-Shack wavefront system in a single measurement (Topcon KR-1 W, Topcon Corp., Tokyo, Japan). This device uses a near-infrared laser beam with a wavelength ranging from 820 to 840 nm to assess the HOAs. The integrated Placido disk technology of the device can measure the corneal radius of curvature up to 0.01 mm. To achieve large physiologic pupil dilation, HOAs were measured in low mesopic conditions in a dark room. The patient was instructed to fixate on a low brightness target during the entire acquisition procedure [18]. All measurements were obtained after blinking to reduce tear film-related HOAs deterioration. The Topcon KR-1 W has an autoalignment and autoacquisition system that removes variability arising from manual focus and alignment. However, the same observer always performed the HOAs measurement to eliminate any possible interobserver variability. Three consecutive HOAs measurements were performed serially just after blinking each time, and the average was computed for analysis. For measuring ocular HOAs, the KR-1 W system is based on the Hartmann-Shack wavefront sensor in which Zernike polynomial expansions up to fourth radial order are provided for a 4- and 6-mm pupil. For measuring anterior corneal monochromatic HOAs, this device utilizes Placido disk technology. Then, aberrations of the internal surfaces were obtained by direct subtraction of the ocular and corneal wavefront data. The following parameters of anterior corneal, internal and ocular wavefronts were obtained for a 6-mm pupil: total HOAs root mean square (RMS), third-order RMS, fourth-order RMS, primary coma RMS (computed for the Zernike terms Z_3_^1^ and Z_3_^− 1^), trefoil RMS (computed for the Zernike terms Z_3_^3^ and Z_3_^− 3^), tetrafoil RMS (computed for the Zernike terms Z_4_^4^ and Z_4_^− 4^), and the primary spherical aberration (Zernike coefficient Z_4_^0^). All RMS values were expressed in micrometres. All patients also underwent a dual Scheimpflug tomography (Galilei G4, Ziemer Ophthalmic Systems AG, Switzerland) evaluation to obtain lens densitometry values. Ocular biometry (IOLMaster, Carl Zeiss Meditec, Dublin, CA, USA) also was performed to acquire axial length measurements. To minimize the effect of diurnal variations of the HOAs, all examinations were performed between 11:00 AM to 2:00 PM.

### Statistical analysis

The analysis was performed using R Statistical Software (version 3.1.3; R Foundation for Statistical Computing, Vienna, Austria). To assess differences in the HOAs among the groups, one-way analysis of covariance was performed. The covariates were age for corneal or ocular HOAs and lens densitometry when internal HOAs were analyzed. When an interaction was observed between the independent variable and the covariate, the sample was stratified into two groups according to the median value of the covariate. Then, one-way analysis of variance was performed for each stratum. Levene’s test was used to check the assumption of homogeneity of variance. Tukey’s test was used to test all possible pairwise differences.

To evaluate the relationship between axial length and HOAs, a partial correlation coefficient was used. Age was selected as a covariate for corneal and ocular HOAs and lens densitometry for internal HOAs. A bootstrap 95% confidence interval then was calculated for this partial correlation coefficient from 5000 bootstrap samples. A *p* value ≤0.05 was considered statistically significant.

## Results

A total of 95 eyes of 95 participants having a mean age of 42.7 ± 11.9 (range, 22 to 65) years were analysed. Low-moderate myopia, no-staphyloma HM, inferior staphyloma, no-dome-shaped macula and dome-shaped macula posterior staphyloma groups were comprised of 31 (20 females, 11 males), 18 (8 females, 10 males), 14 (11 females, 3 males), 15 (11 females, 4 males), and 17 (13 females, 4 males) eyes, respectively. Their mean age was 35.1 ± 11.1, 34.4 ± 8.1, 52.1 ± 7,8, 52.5 ± 6.0 and 49.2 ± 6.5, respectively. The mean spherical equivalent refractions were − 2.1 ± 1.9, − 7.7 ± 1.2, − 11.0 ± 6.5, − 14.5 ± 7.2, and − 13.4 ± 7.8 D, respectively, and the respective mean lens densitometry values were 4.71 ± 1.27%, 4.83 ± 1.62%, 8.29 ± 2.64%, 8.20 ± 3.23%, and 7.59 ± 2.40%. The mean axial lengths were 24.25 ± 1.13, 26.59 ± 1.11, 29.03 ± 2.62, 29.85 ± 1.86, and 29.41 ± 3.34 mm, respectively.

### HOAs

#### Five-group assessment

Corneal, internal, and ocular HOAs data obtained for each of the five groups can be found in Table A1 (see Additional file 1).

In this five-group assessment, significant differences in the anterior corneal tetrafoil, primary spherical aberration, and fourth-order aberrations for the anterior corneal HOAs were found. However, no differences in other anterior corneal, internal or ocular HOAs evaluated were observed.

**Fig. 1:**
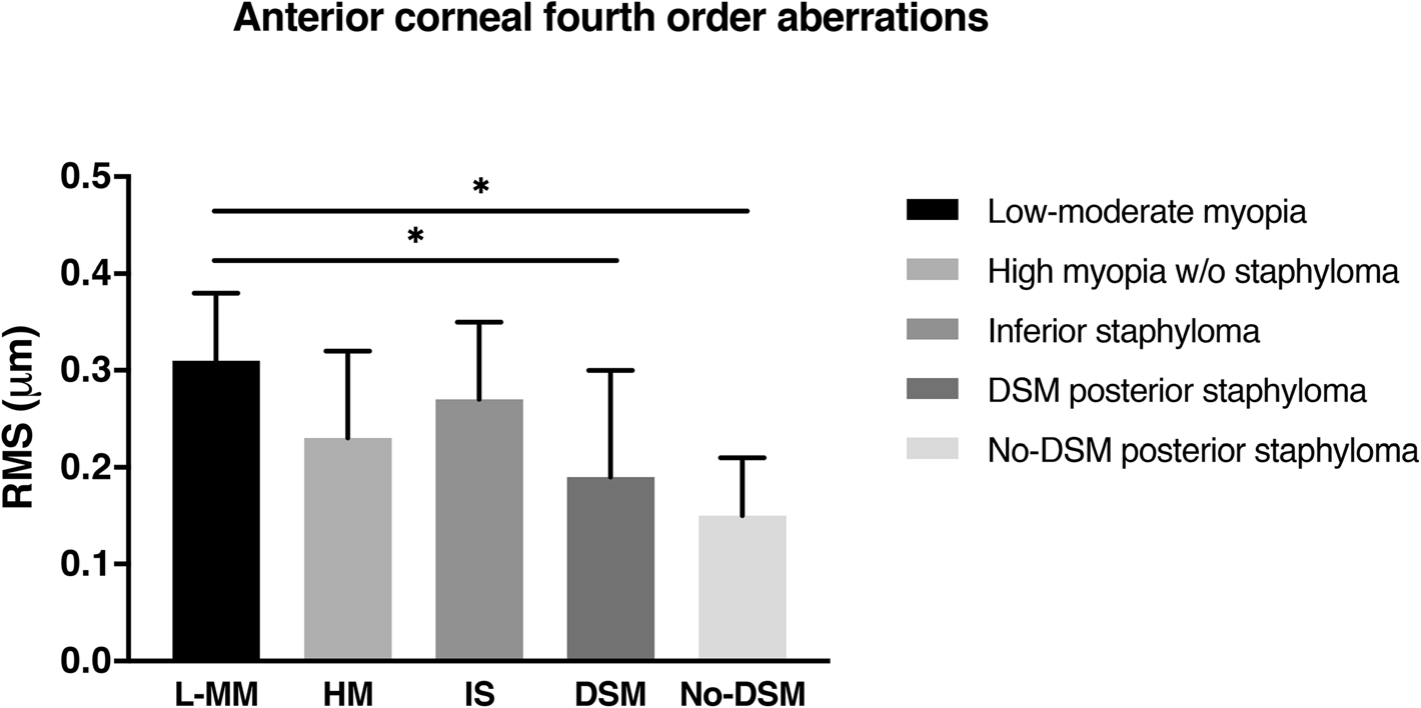
Anterior corneal fourth-order aberrations in patients 45 years of age and younger. w/o, without. DSM, dome-shaped macula

Regarding the anterior corneal fourth-order aberrations, the interaction between group and age was significant (*p* = 0.003). Thus, the entire sample was classified further into two age groups depending on the median age of the sample, ≤45 years and > 45 years old. The younger group was the only one exhibiting significant (*p* = 0.001) differences in the anterior corneal fourth-order root mean square values among the five groups (Fig. 1). The mean differences between the low-moderate myopia group and posterior staphyloma with dome-shaped macula (0.12 μm, 95% confidence intervals, 0.05, 0.19) and no-dome-shaped macula (0.16 μm, 95% confidence intervals, 0.07, 0.24) group were significant in both cases (*p* = 0.05 and *p* = 0.02, respectively).

The group effect was significant (*p* = 0.03) for the anterior corneal tetrafoil aberration. Table 1 shows the anterior corneal tetrafoil values estimated by the model. Significant differences were detected (− 0.12 μm, 95% confidence intervals, − 0.21, − 0.03) in the estimated anterior corneal tetrafoil values between both posterior staphyloma groups (no-dome-shaped macula vs. dome-shaped macula) (*p* = 0.04).

**Table 1:** Estimated anterior corneal tetrafoil values for the five groups evaluated

Group	Estimated mean tetrafoil (μm)	95% CI for the estimated mean (μm)
Low-moderate myopia	0.08	0.03, 0.13
High myopia without staphyloma	0.08	0.02, 0.15
High myopia with inferior staphyloma	0.08	0.01, 0.15
High myopia with No-DSM posterior staphyloma	0.06 ^a^	0.00, 0.13
High myopia with DSM posterior staphyloma	0.18 ^a^	0.12, 0.25

*CI* confidence interval, *DSM* dome-shaped macula

^a^Statistically significant differences between both groups (*p* = 0.04)

**Fig. 2:**
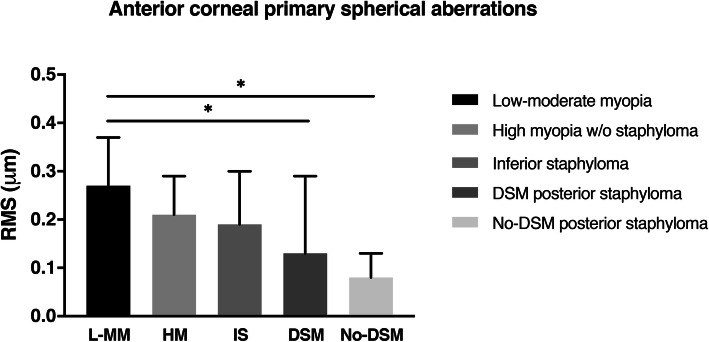
Anterior corneal primary spherical aberration in patients 45 years of age and younger. w/o, without. DSM, dome-shaped macula

Regarding the anterior corneal primary spherical aberration, the interaction between groups was affected significantly (*p* = 0.002) by age; thus, a further sub-classification by age was performed. The younger sub-population (≤45 years) exhibited significant differences (*p* = 0.005) in the anterior corneal primary spherical aberration values among the five groups (Fig. 2). Interestingly, further analysis showed significant differences between the low-moderate myopia group and posterior staphyloma (*p* = 0.05 and *p* = 0.02, respectively) with (0.13 μm, 95% confidence intervals, 0.03, 0.24) and without dome-shaped macula (0.19 μm, 95% confidence intervals, 0.07, 0.31).

#### Two-group assessment (no-staphyloma vs. staphyloma groups)

The HOA data obtained when the entire sample was classified based on the presence or absence of staphyloma (2 groups) are shown in Table A2 (see Additional file 2).

The group effect was significant for the anterior corneal and internal primary spherical aberration. Mean anterior corneal primary spherical aberration for the staphyloma group (0.15 μm, 95% confidence intervals, 0.10, 0.20) was significantly (*p* = 0.004) lower than the no-staphyloma group (0.27 μm, 95% confidence intervals, 0.22, 0.32). Conversely, the estimated mean internal primary spherical aberration value for the staphyloma group (0.04 μm, 95% confidence intervals, − 0.02, 0.11) was significantly (*p* = 0.04) higher than in the no-staphyloma group (− 0.06 μm. 95% confidence intervals, − 0.12, 0.00).

No significant (*p* > 0.05) differences were seen between the no-staphyloma and staphyloma groups for anterior corneal, internal, and ocular total HOAs; trefoil; coma; third- and fourth-order aberrations; and ocular primary spherical aberration.

### Axial length and HOAs

No significant (*p* > 0.05) differences in the axial length were observed among the three former staphyloma groups (HM eyes with inferior or posterior staphyloma groups). Statistically significant (*p* ≤ 0.05) relationships between axial length and HOAs among the five groups are shown in Table 2. Partial correlations coefficients showed that corneal and ocular HOAs can vary differently with increasing axial length depending on each myopia group. However, variation of internal HOAs with increasing axial length was only significant (*p* ≤ 0.05) for coma aberration in the high myopia without staphyloma group, and for primary spherical aberration in both posterior staphyloma groups.

**Table 2:** Significant relationships between axial length and corneal, ocular, and internal aberrations in the five groups studied

Group	Aberration	Corneal HOA	Ocular HOA	Internal HOA
		PCC (95% CI)	PCC (95% CI)	PCC (95% CI)
**Low-moderate myopia**				
	Fourth order	**−0.67 (− 0.85/− 0.42)**	−0.28 (− 0.56/0.01)	−0.15 (− 0.43/0.15)
	Primary SA	**− 0.59 (− 0.78/− 0.45)**	**−0.35 (− 0.62/− 0.04)**	0.09 (− 0.19/0.41)
**High myopia without staphyloma**				
	Total HOAs	0.06 (− 0.52/0.54)	**0.46 (0.14/0.72)**	0.28 (− 0.02/0.70)
	Third order	0.25 (−0.24/0.70)	**0.55 (0.28/0.81)**	0.30 (−0.03/0.68)
	Coma	0.35 (−0.17/0.75)	**0.57 (0.22/0.85)**	**0.45 (0.06/0.79)**
**High myopia with inferior staphyloma**				
	Fourth order	**−0.69 (− 0.90/− 0.29)**	−0.14 (− 0.55/0.48)	−0.02 (− 0.40/0.56)
	Trefoil	**−0.41 (− 0.82/− 0.14)**	−0.13 (− 0.50/0.25)	−0.15 (− 0.63/0.30)
	Primary SA	**−0.78 (− 0.96/− 0.32)**	−0.14 (− 0.55/0.42)	0.01 (− 0.41/0.46)
**High myopia with No-DSM posterior staphyloma**				
	Total HOAs	0.09 (− 0.44/0.62)	**0.60 (0.08/0.89)**	0.20 (−0.59/0.78)
	Fourth order	0.08 (−0.61/0.65)	**0.55 (0.22/0.88)**	0.29 (−0.38/0.83)
	Coma	0.14 (−0.35/0.65)	**0.56 (0.00/0.90)**	0.45 (−0.32/0.82)
	Tetrafoil	−0.40 (− 0.79/0.34)	**0.38 (0.03/0.80)**	− 0.31 (− 0.73/0.64)
	Primary SA	0.14 (− 0.63/0.69)	**0.52 (0.19/0.85)**	**0.58 (0.12/0.87)**
**High myopia with DSM posterior staphyloma**				
	Primary SA	**−0.58 (− 0.88/− 0.15)**	−0.28 (− 0.77/0.24)	**0.49 (0.00/0.76)**

The table shows only the cases in which statistically significant associations between axial length and higher order aberrations were found for each group. *HOAs* higher order aberrations, *PCC* partial correlation coefficient, *SA* spherical aberration, *CI* confidence interval, *DSM* dome-shaped macula. Values in bold indicate significant (*p* ≤ 0.05) associations

## Discussion

Wavefront sensors were initially utilized for corneal refractive surgery in the clinical setting. This technology is now also widely used for phakic and pseudophakic intraocular lens or intracorneal ring implantations. Meticulous characterization of HOAs in HM may assist in a better understanding of the visual characteristics of patients with HM and to improve the optical customized designs of intraocular lens to accurately restore vision in highly myopic eyes. Eyes with posterior staphyloma tended to have lower anterior corneal spherical aberration in adults younger than 45 years. Simultaneous presence of dome-shaped macula also resulted in higher anterior corneal tetrafoil aberrations. Less positive anterior corneal primary spherical aberration and less negative internal primary spherical aberration should be expected in highly myopic eyes with staphyloma.

Age-related changes in HOAs have been previously reported [3, 5, 6]. Corneal third-order aberrations have been reported to increase in aging eyes [6]. Internal fourth-order aberrations, which are mainly affected by changes in the shape of the lens, also tend to increase with age [5, 6]. Taken this into account, in this study a statistical analysis that included both age and lens as covariates was performed. Patient age is easily determined; however, because lens aging is not that easily objectively assessed using slit-lamp biomicroscopic imaging, lens densitometry data provided by a dual Scheimpflug imaging system was also recorded.

There is great controversy in the literature regarding not only the possible differences in monochromatic HOAs among hyperopia, emmetropia and myopia, but also which HOAs might differ. On the one hand, several authors have reported no differences in the magnitude of the HOAs with different refractive errors, even in a young population [19–21]. However, another study that assessed ocular aberrations reported significant higher total HOAs values in myopic eyes compared with emmetropic and hyperopic eyes [22]. The internal and, therefore, the total ocular HOAs are age-dependent [3, 5, 6]; thus, differences among studies should be expected if the patients’ ages vary. It also is important to consider the pupil size in for each study because HOAs increase with larger pupils and all aberrations are not equally affected [23]. Moreover, great variability in HOAs has already been reported among healthy individuals, where the standard deviation for each Zernike aberration term (above the second order) usually is larger than its corresponding mean [24]. Another consideration is that different studies have used different wavefront sensors and the reliability may differ when obtaining the HOAs [25]. In case of Topcon KR-1 W system, ocular and the anterior corneal monochromatic HOAs area measured using Hartmann-Shack and Placido disk technology, respectively, while internal ones are calculated after subtracting the anterior corneal HOAs from whole eye wavefront data. This procedure has limitations because the accuracy of Placido-disk based devices when providing HOAs has been reported to be restricted [26]. Nonetheless, combining ocular and anterior corneal HOAs provides a unique opportunity for understanding the optics of the eye for clinical purposes. Consequently, comparing outcomes among studies is difficult because of the variability of the samples. Besides, regarding the HOAs variability observed in the present study, staphyloma eyes showed higher variability than low-moderate myopia eyes for most of the HOAs analysed (Additional files 1 and 2). Thus, some differences in HOAs among groups could have not reached statistical significance because of the high inter-individual variability, and also because of the sample size recruited for each group, which might not have been enough large to detect these possible differences.

In the present study we found significant differences in monochromatic HOAs among the five study groups in the anterior corneal HOAs and particularly in younger participants. Patients with posterior staphyloma regardless of the presence of dome-shaped macula had lower corneal fourth-order and spherical aberration values if they were younger than 45 years compared with the patients in the low-moderate myopia group (Figs. 1, 2). However, in the posterior staphyloma group with dome-shaped macula the anterior corneal tetrafoil value was higher (**≈** 10 μm) compared with the other groups studied (Table 1). Considering that the current axial lengths were similar (**≈** 29 mm) among the eyes with inferior and posterior staphyloma, it seems that the corneal profile of the eyes with posterior staphyloma might change differently compared with eyes with inferior staphyloma. In case of posterior staphyloma, the posterior axial elongation of the eye could also associate uniform traction of the anterior segment, specifically in the scleral spur area, which could also modify the corneal shape. This biomechanical traction is likely to affect equally the whole cornea in case of posterior staphyloma (in contrast to inferior staphyloma), resulting in a homogeneous reduction of the corneal asphericity (more negative asphericity values). This asphericity reduction is likely to be responsible for the decrease in the anterior corneal primary spherical aberration observed in the posterior staphyloma eyes (in comparison with low-moderate myopia eyes) (Fig. 2). Moreover, the spherical aberration is a fourth-order aberration, thus, its change might have contributed also to find differences in anterior corneal fourth-order aberrations in both posterior staphyloma groups.

When the participants were subdivided into two groups based on the presence or absence of staphyloma, we also observed that longer eyes tended to have lower estimated anterior corneal spherical aberration (less positive) and higher internal spherical aberration (less negative). In fact, the internal spherical aberration was no longer negative on average, as usually occurs in eyes with axial length in the normal range [13, 27]. The variable that clearly differed between these two subgroups was the axial length, which resulted in a noticeable change in the anterior corneal and internal spherical aberration, once the effects of age and lens interaction were removed. Nonetheless, the decreased anterior corneal spherical aberration was compensated by a less negative internal spherical aberration; thus, no significant variation in the resulting ocular spherical aberration was observed compared with the myopic eyes without staphyloma. Only one previous study also reported the relationship between HOAs and eyes with staphyloma in phakic patients. Kasahara et al. [10] performed a multiple regression analysis and evaluated eyes with high myopia (mean refractive error, − 13.5 D; mean axial length, 29.4 mm) similar to our sample. Those authors reported increased total ocular HOAs as a result of less negative internal spherical aberration in highly myopic eyes and reduced corneal spherical aberration as we did. Based on the current outcomes, it seems that even in HM, the optical system tends to be compensated, thus avoiding a great increase in total ocular HOAs as occurs in normal eyes. It was previously reported that central corneal curvature (keratometry) does not necessarily change with increasing axial length in HM eyes after several years of follow-up [28]. Consequently, it is likely that in the current highly myopic eyes, a change in the corneal profile occurred that reduced the positive corneal spherical aberration typically found in normal eyes, particularly those with posterior staphyloma.

We also evaluated the relationship between axial length and HOAs after removing confounding factors such as age and lens aging (Table 2). A significant relationship between longer axial length and higher internal primary positive spherical aberration in posterior staphyloma groups was observed, similar to was reported by Kasahara et al. [10] Those authors also reported a direct relationship between the degree of myopia and ocular primary spherical aberration. However, we only observed this finding in the posterior staphyloma group without a dome-shaped macula. In fact, it was noteworthy that our dome-shaped macula posterior staphyloma group did not show a significant relationship between the axial length and ocular HOAs. It seems that the bulging from the dome-shaped macula tended to compensate for the elongation of the eye, which reduced the ocular HOAs.

Outcomes presented in this manuscript should be considered when evaluating patients with high myopia and staphyloma who may be candidates for refractive surgery. Phakic intraocular lenses implantation is usually recommended in patients with high myopia who are not good candidates for excimer laser surgery, especially since the new phakic intraocular lens models (ICL, STAAR Surgical, Monrovia, California, USA) with a higher vault and central aperture are now available [29, 30]. These phakic intraocular lens has negative spherical aberration and its magnitude is higher (i.e., more negative values) in the intraocular lenses with higher negative power [30, 31]. Consequently, keeping in mind that eyes with high myopia and staphyloma may have reduced positive ocular spherical aberration [10] and that phakic intraocular lens induce negative spherical aberration [31, 32], implantation will increase the internal negative spherical aberration resulting in a postoperative increase of ocular HOAs. This limitation in the postoperative optical quality in patients with high myopia and staphyloma can be worsened if large corneal incisions (> 3.2 mm) are created intraoperatively, as previously reported by Pérez-Vives et al. [32] Refractive surgeons should be aware of this possible postoperative scenario when counselling these patients. Phakic intraocular lens manufacturers also should be aware of the current and previous study outcomes [10] to facilitate design of new spherical aberration-free phakic intraocular lens as with pseudophakic ones. Thus, patients with high myopia and staphyloma will benefit from this surgery as much as patients with moderate myopia already do.

The major limitation of the current study was that we did not use cycloplegic drugs when measuring the HOAs. It has been reported that ocular primary spherical aberration becomes more negative with increasing accommodation; however, a substantial ratio between the magnitude of accommodation and change in spherical aberration was observed only in subjects older than 50 years [33]. At the same time, this population is unlikely to perform unconscious accommodation when targeting pseudo-distance objects, because the ability to accommodate is already reduced. Thus, we think that the HOAs that we measured are similar to the actual HOAs that patients had. Another limitation of the study might be the calculated sample size because we selected a large effect size for calculations. HOAs might show high inter-individual variability [24, 25], thus, larger sample sizes could help to better characterize staphyloma eyes in terms of HOAs. However, our study design was able to show significant differences in HOAs between low-moderate myopia eyes and posterior staphyloma eyes with or without dome-shaped macula, and also between the two later groups. Nonetheless, these outcomes should be confirmed in future studies including larger sample sizes. Another limitation is that refractive error and axial length were not used as covariates when performing the statistical analysis, thus, some of the findings observed could be related to the differences in both variables among groups. However, we did not include both variables as covariates because first, there were not significant differences in axial length among the staphyloma groups (inferior and posterior ones), and second, if we have included them, the statistical models would have been more complex, and an overfitting problem could have occurred. Finally, random selection of one eye when both eyes of one patient met the criteria was performed to avoid bias [34]. In contrast, the presence of enantiomorphism in HOAs [35] was not considered when selecting the eye data computed for analysis, thus, it can be considered a limitation of the study. However, the most remarkable finding of the study is related to primary spherical aberration, and enantiomorphism does not affect this HOA.

## Conclusions

In conclusion, presence of posterior staphyloma is associated with reduced anterior corneal spherical aberration in younger adults (< 45 years), while a concomitant dome-shaped macula in these HM eyes will result in increased anterior corneal tetrafoil values. Besides, HM eyes, regardless of the presence of dome-shaped macula and the staphyloma location, are likely to have lower anterior corneal primary spherical aberration (less positive) and less negative internal primary spherical aberration compared with myopic eyes without staphyloma. However, the balance between corneal and internal HOAs, typically observed in normal eyes, seems to be maintained in eyes with high myopia and staphyloma. Furthermore, to some extent, the cornea appears to mimic in a specular fashion the profile of the posterior pole in eyes with high myopia and staphyloma. Interestingly, while bulging of the posterior pole in eyes with staphyloma seems to be a continuous progressive process that involves the posterior sclera [36, 37], it is not necessarily associated with a parallel corneal profile change (less positive spherical aberration) over the years, as seen in the current patients over 45 years. This finding suggests a potential different behaviour of the cornea in adults over 45 years. The current data also may be helpful for clinicians and researchers, because an increasing number of refractive surgery candidates with long axial length undergoing either laser excimer or intraocular surgery [15, 38, 39], demand better quality of vision and need adequate counselling before a customized refractive procedure.

## Supplementary Information

**Additional file 1: Table A1.** Higher order aberration values obtained for the five myopia groups evaluated.

**Additional file 2: Table A2.** Higher order aberration values obtained for no-staphyloma and staphyloma eyes.

## Data Availability

The datasets used and/or analysed during the current study are available from the corresponding author on reasonable request.

## Abbreviations

D: diopters
HM: high myopia
HOA: higher order aberrations
