# Supplementary material for: Monochromatic higher order aberrations in highly myopic eyes with Staphyloma
Source: BMC Ophthalmol. 2021 May 18;21:223. doi: 10.1186/s12886-021-01965-9 (PMC8130529; doi:10.1186/s12886-021-01965-9)
Supplement: Supplementary file 2 — Additional file 2: Table A2. Higher order aberration values obtained for no-staphyloma and staphyloma eyes. [file 12886_2021_1965_MOESM2_ESM.docx]

SUPPLEMENTARY MATERIAL. TABLE A2.

Manuscript title:

Monochromatic Higher Order Aberrations in Highly Myopic Eyes with Staphyloma.

Santiago Delgado-Tirado, MSc^*1^; Alberto López-Miguel, PhD^*^ †^1^; Yazmin Báez-Peralta, MSc^*^; Lucía González-Buendía, MSc^*^; Itziar Fernández, PhD*; Jorge L. Alió, PhD †§; Miguel J. Maldonado, PhD* † ; Rosa M. Coco-Martín, PhD* † .

^*^ Instituto de Oftalmobiología Aplicada (IOBA), Universidad de Valladolid, Valladolid, Spain.

† Networks for Cooperative Research in Health (Oftared); Instituto de Salud Carlos III, Madrid, Spain.

§ Division of Ophthalmology, School of Medicine, Universidad Miguel Hernández, Alicante, Spain.

1. Both authors contributed equally to the work.

| **Table A2.** Higher order aberration values obtained for no-staphyloma and staphyloma eyes. | | | | | | | | | | | | | | | | | | | | | | | | |
| --- | --- | --- | --- | --- | --- | --- | --- | --- | --- | --- | --- | --- | --- | --- | --- | --- | --- | --- | --- | --- | --- | --- | --- | --- |
| **Corneal** | | | | | | | | | | | | | | | | | | | | | | | | |
| Group | Total (RMS) (µm) | | | | | | 3rd order (RMS) (µm) | | | | | | 4th order (RMS) (µm) | | | | | | Trefoil (RMS) (µm) | | | | | |
|  | Mean | ± SD | 95% CI | | Range | | Mean | ± SD | 95% CI | | Range | | Mean | ± SD | 95% CI | | Range | | Mean | ± SD | 95% CI | | Range | |
|  |  |  | Inf. | Sup. | Min. | Max. |  |  | Inf. | Sup. | Min. | Max. |  |  | Inf. | Sup. | Min. | Max. |  |  | Inf. | Sup. | Min. | Max. |
| No-staphyloma | 0.41 | 0.18 | 0.35 | 0.46 | 0.12 | 1.29 | 0.25 | 0.19 | 0.20 | 0.31 | 0.09 | 1.24 | 0.28 | 0.08 | 0.26 | 0.30 | 0.04 | 0.45 | 0.15 | 0.11 | 0.12 | 0.18 | 0.02 | 0.70 |
| Staphyloma | 0.50 | 0.46 | 0.37 | 0.64 | 0.09 | 2.85 | 0.37 | 0.29 | 0.29 | 0.46 | 0.08 | 1.31 | 0.28 | 0.27 | 0.20 | 0.36 | 0.03 | 1.70 | 0.23 | 0.24 | 0.16 | 0.30 | 0.02 | 1.10 |
|  | | | | | | | | | | | | | | | | | | | | | | | | |
| Group | Coma (RMS) (µm) | | | | | | Tetrafoil (µm) | | | | | | Spherical (µm) | | | | | |  | | | | | |
|  | Mean | ± SD | 95% CI | | Range | | Mean | ± SD | 95% CI | | Range | | Mean | ± SD | 95% CI | | Range | |  |  |  |  |  |  |
|  |  |  | Inf. | Sup. | Min. | Max. |  |  | Inf. | Sup. | Min. | Max. |  |  | Inf. | Sup. | Min. | Max. |  |  |  |  |  |  |
| No-staphyloma | 0.19 | 0.17 | 0.15 | 0.24 | 0.03 | 1.03 | 0.07 | 0.05 | 0.05 | 0.08 | 0.00 | 0.22 | 0.24 | 0.09 | 0.22 | 0.27 | -0.01 | 0.39 |  |  |  |  |  |  |
| Staphyloma | 0.25 | 0.21 | 0.19 | 0.31 | 0.02 | 0.88 | 0.13 | 0.18 | 0.08 | 0.19 | 0.01 | 0.86 | 0.18 | 0.18 | 0.13 | 0.24 | -0.10 | 0.96 |  |  |  |  |  |  |
|  | | | | | | | | | | | | | | | | | | |  |  |  |  |  |  |
| **Internal** | | | | | | | | | | | | | | | | | | | | | | | | |
| Group | Total (RMS) (µm) | | | | | | 3rd order (RMS) (µm) | | | | | | 4th order (RMS) (µm) | | | | | | Trefoil (RMS) (µm) | | | | | |
|  | Mean | ± SD | 95% CI | | Range | | Mean | ± SD | 95% CI | | Range | | Mean | ±SD | 95% CI | | Range | | Mean | ± SD | 95% CI | | Range | |
|  |  |  | Inf. | Sup. | Min. | Max. |  |  | Inf. | Sup. | Min. | Max. |  |  | Inf. | Sup. | Min. | Max. |  |  | Inf. | Sup. | Min. | Max. |
| No-staphyloma | 0.29 | 0.15 | 0.24 | 0.33 | 0.06 | 0.87 | 0.20 | 0.11 | 0.17 | 0.23 | 0.04 | 0.6 | 0.16 | 0.10 | 0.13 | 0.19 | 0.03 | 0.55 | 0.12 | 0.10 | 0.09 | 0.15 | 0.03 | 0.60 |
| Staphyloma | 0.43 | 0.45 | 0.29 | 0.56 | 0.07 | 2.61 | 0.30 | 0.32 | 0.21 | 0.40 | 0.06 | 1.62 | 0.23 | 0.25 | 0.16 | 0.30 | 0.02 | 1.23 | 0.15 | 0.23 | 0.08 | 0.22 | 0.01 | 1.21 |
|  | | | | | | | | | | | | | | | | | | | | | | | | |
| Group | Coma (RMS) (µm) | | | | | | Tetrafoil (µm) | | | | | | Spherical (µm) | | | | | |  | | | | | |
|  | Mean | ± SD | 95% CI | | Range | | Mean | ± SD | 95% CI | | Range | | Mean | ± SD | 95% CI | | Range | |  |  |  |  |  |  |
|  |  |  | Inf. | Sup. | Min. | Max. |  |  | Inf. | Sup. | Min. | Max. |  |  | Inf. | Sup. | Min. | Max. |  |  |  |  |  |  |
| No-staphyloma | 0.15 | 0.09 | 0.13 | 0.18 | 0.02 | 0.45 | 0.07 | 0.06 | 0.05 | 0.08 | 0.00 | 0.35 | -0.06 | 0.13 | -0.10 | -0.03 | -0.30 | 0.46 |  |  |  |  |  |  |
| Staphyloma | 0.23 | 0.25 | 0.15 | 0.30 | 0.01 | 1.08 | 0.13 | 0.19 | 0.07 | 0.18 | 0.01 | 0.97 | 0.05 | 0.25 | -0.02 | 0.13 | -0.83 | 0.86 |  |  |  |  |  |  |
|  | | | | | | | | | | | | | | | | | | |  |  |  |  |  |  |
| **Ocular** | | | | | | | | | | | | | | | | | | | | | | | | |
| Group | Total (RMS) (µm) | | | | | | 3rd order (RMS) (µm) | | | | | | 4th order (RMS) (µm) | | | | | | Trefoil (RMS) (µm) | | | | | |
|  | Mean | ± SD | 95% CI | | Range | | Mean | ± SD | 95% CI | | Range | | Mean | ± SD | 95% CI | | Range | | Mean | ± SD | 95% CI | | Range | |
|  |  |  | Inf. | Sup. | Min. | Max. |  |  | Inf. | Sup. | Min. | Max. |  |  | Inf. | Sup. | Min. | Max. |  |  | Inf. | Sup. | Min. | Max. |
| No-staphyloma | 0.39 | 0.24 | 0.32 | 0.46 | 0.06 | 1.11 | 0.30 | 0.21 | 0.24 | 0.36 | 0.04 | 1.06 | 0.20 | 0.15 | 0.16 | 0.24 | 0.01 | 0.82 | 0.18 | 0.16 | 0.13 | 0.22 | 0.01 | 0.87 |
| Staphyloma | 0.61 | 0.45 | 0.48 | 0.75 | 0.07 | 1.92 | 0.47 | 0.36 | 0.36 | 0.57 | 0.04 | 1.45 | 0.35 | 0.29 | 0.26 | 0.43 | 0.05 | 1.25 | 0.20 | 0.19 | 0.15 | 0.26 | 0.03 | 0.89 |
|  | | | | | | | | | | | | | | | | | | | | | | | | |
| Group | Coma (RMS) (µm) | | | | | | Tetrafoil (µm) | | | | | | Spherical (µm) | | | | | |  | | | | | |
|  | Mean | ±SD | 95% CI | | Range | | Mean | ± SD | 95% CI | | Range | | Mean | ± SD | 95% CI | | Range | |  |  |  |  |  |  |
|  |  |  | Inf. | Sup. | Min. | Max. |  |  | Inf. | Sup. | Min. | Max. |  |  | Inf. | Sup. | Min. | Max. |  |  |  |  |  |  |
| No-staphyloma | 0.23 | 0.20 | 0.17 | 0.29 | 0.02 | 0.96 | 0.07 | 0.07 | 0.05 | 0.09 | 0.01 | 0.38 | 0.15 | 0.15 | 0.11 | 0.19 | -0.07 | 0.73 |  |  |  |  |  |  |
| Staphyloma | 0.39 | 0.34 | 0.29 | 0.49 | 0.03 | 1.22 | 0.10 | 0.11 | 0.07 | 0.14 | 0.01 | 0.50 | 0.25 | 0.28 | 0.17 | 0.34 | -0.68 | 0.96 |  |  |  |  |  |  |

SD: standard deviation. CI: confidence interval. RMS: root mean square; Inf.: inferior; Sup.: superior; Min.: minimum; Max.: maximum.
